# Pharmacological management of upper gastrointestinal Crohn’s disease: a systematic review

**DOI:** 10.1093/ecco-jcc/jjaf187

**Published:** 2025-10-28

**Authors:** Mark Chatto, Dimah Alaskar, Christopher Ma, Yuhong Yuan, Sudheer Kumar Vuyyuru, Talat Bessissow, Neeraj Narula, Silvio Danese, Laurent Peyrin-Biroulet, Siddharth Singh, Vipul Jairath, Rocio Sedano

**Affiliations:** Department of Medicine, Division of Gastroenterology, Western University, London, Ontario, Canada; Section of Gastroenterology, Department of Medicine, Makati Medical Center, Manila, Philippines; Department of Medicine, Division of Gastroenterology, Western University, London, Ontario, Canada; Division of Gastroenterology & Hepatology, Department of Medicine, Cumming School of Medicine, University of Calgary, Calgary, Alberta, Canada; Department of Community Health Sciences, University of Calgary, Calgary, Alberta, Canada; Department of Medicine, Division of Gastroenterology, Western University, London, Ontario, Canada; Department of Medicine, London Health Science Center, London, Ontario, Canada; Department of Medicine, Division of Gastroenterology, Western University, London, Ontario, Canada; Division of Gastroenterology and Hepatology, McGill University Health Centre, Montreal, Quebec, Canada; Department of Medicine (Division of Gastroenterology) and Farncombe Family Digestive Health Research Institute, McMaster University, Hamilton, ON, Canada; Department of Biomedical Sciences, Humanitas University, Pieve Emanuele, Milan, Italy; IBD Center, Humanitas Research Hospital, IRCCS, Rozzano, Milan, Italy; Division of Gastroenterology and Hepatology, McGill University Health Centre, Montreal, Quebec, Canada; Department of Gastroenterology, CHRU Nancy, INSERM NGERE, Université de Lorraine, Vandœuvre-lès-Nancy, F-54500, France; Division of Gastroenterology and Hepatology, Department of Medicine, Mayo Clinic Arizona, United States; Department of Medicine, Division of Gastroenterology, Western University, London, Ontario, Canada; Department of Medicine, London Health Science Center, London, Ontario, Canada; Departments of Epidemiology and Biostatistics, Western University, London, Ontario, Canada; Department of Medicine, Division of Gastroenterology, Western University, London, Ontario, Canada; Department of Medicine, London Health Science Center, London, Ontario, Canada; Departments of Epidemiology and Biostatistics, Western University, London, Ontario, Canada; Department of Medicine, St Joseph’s Health Care, London, Ontario, Canada

**Keywords:** UGI, Crohn’s disease, systematic review

## Abstract

**Background:**

Upper gastrointestinal Crohn’s disease (UGICD) is an uncommon phenotype with limited management guidelines. We reviewed evidence on the safety and efficacy of pharmacological interventions for UGICD.

**Methods:**

We searched MEDLINE, Embase, and Cochrane CENTRAL (1990-2025) for randomized controlled trials (RCTs) and comparative observational studies evaluating pharmacological or dietary interventions for UGICD, including esophagus to jejunum. Two reviewers screened studies, extracted data, and assessed bias (Newcastle–Ottawa Scale). Primary outcomes were clinical remission and response. Due to limited, heterogeneous evidence, data are summarized descriptively.

**Results:**

Of 1207 citations, 11 observational studies (nine retrospective, two prospective) and post-hoc analyses from two RCTs met the criteria, involving 387 patients. Most had ileocolonic involvement (280/387; 72.3%); only 8.5% (33/387) had isolated UGICD. Five studies (137 patients) reported esophageal CD. Follow-up ranged from 6 weeks to 28 years. Interventions and outcomes varied. Anti-tumor necrosis factor (anti-TNF) drugs and corticosteroids, alone or combined with other treatments, were associated with improvements in clinical outcomes, endoscopic healing, and histology, but controlled data are lacking. Other therapies, including proton pump inhibitors, H2-receptor antagonists, enteric nutrition, immunomodulators, anti-integrins, and anti-interleukin12/23, showed moderate to minimal improvement.

**Conclusions:**

Our systematic review highlights a paucity of evidence to inform therapeutic strategies in UGICD. Positive outcomes were reported for corticosteroids and anti-TNF, but from small observational and uncontrolled studies. Data for most advanced therapies remain limited, highlighting a large unmet need to inform clinical practice.

## Graphical abstract

**Graphical Abstract:**
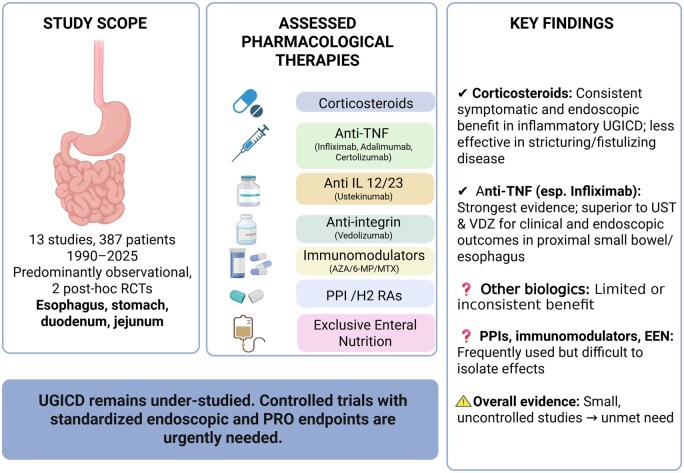

## 1. Introduction

Crohn’s disease (CD) is a chronic inflammatory bowel disease that can affect the entire gastrointestinal (GI) tract, from mouth to anus. The vast majority of interventions for moderate to severe CD have been developed in patients with ileo-colonic CD, defined as within the reach of the colonoscope. Upper GI (UGI) CD has not been precisely defined in the literature.

The prevalence of CD affecting the esophagus, stomach, and duodenum was originally thought to range from 0.5% to 5%.^1^^,^^2^ However, recent studies suggest this may have been an underrepresentation, reporting prevalence rates between 13% and 15%.^3^^,^^4^ Despite its low prevalence, UGI involvement is clinically important: patients may present with atypical symptoms of dyspepsia, epigastric pain, early satiety, vomiting, or UGI bleeding, and these symptoms can overlap with peptic ulcer disease or other common disorders.^5^^,^^6^ Delayed recognition of UGICD can lead to complications such as stricturing disease, penetrating disease, recurrence, hospitalizations, and surgeries, making UGI involvement a negative prognostic factor for the course of CD.^7^^,^^8^

Management of CD is well established and described in multiple guidelines and recommendations, mainly referring to ileal, colonic, and ileocolonic CD. The vast majority of interventions for moderate to severe CD have been evaluated in patients with ileo-colonic CD, defined as within the reach of the colonoscope. In contrast, management of UGICD poses a particular challenge because of lack of consistency in definitions^9^ and paucity of high-level evidence to guide therapy. The mainstay treatments for CD—corticosteroids, immunosuppressants, and biologic agents—are used in UGICDs by extension of their proven efficacy in ileocolonic disease, but no randomized trials have specifically evaluated these interventions in isolated UGI disease.^10^

While previous reviews have broadly examined the prevalence, diagnosis, and overall management strategies for UGICD, none has specifically and systematically evaluated pharmacological interventions with clearly defined efficacy and safety outcomes. The objective of this review is therefore to provide a focused synthesis of existing data on these specific interventions, rigorously assessing their efficacy, safety, and clinical utility, and establish a foundation for future targeted research in this under-studied CD phenotype.

## 2. Methods

### 2.1. Data sources and study selection

The Preferred Reporting Items for Systematic Reviews and Meta-analysis (PRISMA)-2020^11^ guidelines were followed for the conduct and reporting of this systematic review (Supplementary Appendix 1). We systematically searched MEDLINE, Embase, and the Cochrane CENTRAL Register of Controlled Trials (via Ovid) from January 1, 1990, to March 5, 2025, using controlled vocabulary and keywords related to CD and UGI tract involvement (esophagus, stomach, duodenum, jejunum). The detailed search strategies are provided in Supplementary Appendix 2. We excluded non-English articles, conference abstracts, and case reports from the search. References of relevant studies, review articles, and prior meta-analyses were reviewed to identify additional eligible studies.

### 2.2. Study eligibility criteria

Fully published, comparative studies evaluating the efficacy of pharmacological (eg, corticosteroids, immunomodulators, biologics) or dietary interventions for patients with UGICD, defined as involvement of the esophagus, stomach, duodenum, or jejunum, were eligible for inclusion. Eligible studies included randomized controlled trials (RCTs) (including post-hoc analysis of an RCT), and observational studies that directly compared at least two active interventions or compared an active intervention to placebo or no treatment. Studies were required to include at least 10 patients with UGICD. There was no age limitation.

We defined UGICD as CD involving the esophagus, stomach, duodenum, or jejunum. The inclusion of jejunal disease was intentional, based on its phenotypic and prognostic similarity to other proximal locations and its association with a more aggressive disease course. In studies that reported jejunal involvement, investigators explicitly distinguished jejunal from proximal ileal lesions using endoscopic or radiologic definitions. For example, Huang et al. used balloon-assisted enteroscopy to define the jejunum as distal to the duodenum and separate from the proximal ileum. In our synthesis, we retained these study-level definitions for transparency but applied a pragmatic anatomy-based framework, considering the jejunum to extend from the ligament of Treitz to the jejuno-ileal junction, supplemented by radiologic correlation where necessary.

Studies were excluded if they (1) enrolled fewer than 10 patients with UGICD; (2) included only patients with terminal ileum or proximal ileum CD; (3) did not report outcomes specifically for UGICD patients; (4) lacked clear reporting of clinical outcomes after pharmacological or dietary interventions; (5) primarily evaluated surgical or endoscopic interventions; (6) were published in languages other than English; (7) were published only as conference abstracts; or (8) were published before 1990. The year 1990 was selected to reflect the contemporary interventions of inflammatory bowel disease (IBD). For retrospective studies that did not clearly define their study design, or were mislabeled as “case series” by primary authors, if they met the inclusion criteria, they were classified as retrospective cohort studies, according to Dekkers et al.^12^

### 2.3. Outcome assessment and data extraction

Articles were independently assessed by two authors [MC, RS, YY, or DA] according to predefined eligibility criteria. Disagreements were resolved through consensus or consultation with a third reviewer [YY or RS]. Titles and abstracts were screened, and the full texts of potentially eligible studies were retrieved for detailed evaluation. Data extraction was performed independently by two authors [MC, DA] using a standardized form in a Microsoft Excel spreadsheet [Office 365 edition; Microsoft], and discrepancies were resolved by discussion or a third author [YY or RS].

Efficacy outcomes extracted included the proportion of patients achieving clinical remission (primary outcome), clinical response, endoscopic remission, endoscopic improvement, histological improvement or remission, radiological improvement or remission, and changes in quality-of-life measures (secondary outcomes). Safety outcomes included the proportion of patients experiencing adverse events (primary safety outcome), serious adverse events, and infections (secondary safety outcomes).

Extracted trial characteristics included study design (eg, RCT, observational cohort), year of publication, country of study, number of participants, number of UGICD patients, duration of follow-up, intervention details (type and route of pharmacological or dietary interventions, dosing schedule, concomitant medications), comparator group (active treatment, placebo, or no treatment), and time points of outcome assessment. Participant characteristics extracted included age, sex, disease duration, and prior treatment history.

### 2.4. Data synthesis and statistical methods

Given the anticipated heterogeneity and limited data from the included studies, a descriptive synthesis was performed. Data were summarized qualitatively according to type of intervention (pharmacological or dietary) and reported outcomes, including clinical remission, clinical response, endoscopic remission, endoscopic improvement, histological improvement, radiological improvement, and safety outcomes.

Stratified descriptive summaries were planned based on intervention type, disease location (esophagus, stomach, duodenum, jejunum), study design, patient characteristics, and outcome definitions to qualitatively explore sources of variability among studies. Due to the limited number of studies and substantial heterogeneity in populations, interventions, and reported outcomes, quantitative meta-analysis was not feasible.

### 2.5. Risk of bias assessment

The methodological quality and risk of bias of observational studies were assessed using the Newcastle–Ottawa Scale (NOS),^13^ evaluating each study based on participant selection criteria, comparability of groups, and outcome ascertainment. Scores were summarized descriptively in tables and narratively within the paper.

Two authors [MC, DA] independently assessed the methodological quality of the included observational studies. Items evaluated included participant selection criteria, comparability of study groups, and ascertainment of outcomes, with scores ranging from 0 to 9 points. Studies scoring ≥7 points were considered of high methodological quality, scores of 4–6 were considered moderate quality, and scores ≤3 indicated low methodological quality. Any discrepancies between reviewers were resolved through discussion or by consultation with a third reviewer [YY or RS]. Post-hoc analysis of an RCT was considered as a prospective cohort study when assessing the risk of bias, because the randomization was not stratified by disease location. Results of the NOS assessments are summarized in Table 1.

**Table 1.** Study characteristics.

Author name [Trial], (year), country	Study design, duration of follow-up^a^	No. of centers	Population of study; no. with UGICD (other concomitant CD location)	Method of diagnosis of UGICD; inclusion
**D’Haens et al.^25^ [DIVERGENCE 1] (2023), Belgium, USA, Austria, Canada, Czechia, Ukraine, Spain, Italy, Hungary, Germany, France, UK**	RCT, 24 weeks	Multi-center	78 adult CD, 17 with jejunal ulcerations, (61/78 had terminal ileum involvement, 24/78 with distal ileum ulcers)	Radiologic (active inflammation in at least 1 SB segment on MRE (segmental MaRIA [MaRIAseg] 7); and prior failure of corticosteroids, immunomodulators, TNF, vedolizumab, or UST; clinical (moderate-severe CDAI)
**D’Haens et al. (1994)^14^, Belgium**	Retrospective case series observational, 12 years	Single center	14 adult CD, all with esophageal (all 14 had concomitant terminal ileum and colorectal lesions)	Typical endoscopic ± histologic lesions in esophagus, excluding other causes
**Miehsler et al. (2001)^15^, Austria**	Retrospective case series observational, 34 months	Single center	32 adult CD, all UGICD up to duodenum (all 32 had ileal or ileocolonic CD)	Endoscopic, histologic, radiologic; all patients taken from those who have had the sucrose–lactose mannitol test
**Olbjorn et al. (2014)^23^, Norway**	Prospective cohort, 2 years	Multi-center	36 pediatric CD, 16 with CD-specific UGI lesions	Endoscopic, clinical, histologic; exclude *Helicobacter pylori*, celiac
**De Felice et al. (2015)^6^, USA**	Retrospective case series observational, 18 years	Single center	24 adult CD, all esophageal (23 had extraesophageal CD, 3 perianal fistulizing CD)	Endoscopic, histologic, clinical
**Lambin et al. (2020)^18^, France, Belgium**	Retrospective cohort observational, 5 years	Multi-center, 5 years	60 adult CD, all with UGI stricture (12 isolated UGICD, 23 with concomitant ileal involvement, 5 colonic, 20 both ileal and colonic)	Endoscopic, clinical (symptomatic UGICD stricture, not passable by endoscopy)
**Huang et al. (2025)^22^, China**	Retrospective observational cohort, 1 year	Single center, 1 year	110 adult small bowel CD, 39 with jejunal involvement (103 proximal ileum)	Endoscopic—balloon assisted. Enteroscopy; jejunum defined as “after duodenum & prior to proximal ileum”; proximal ileum (30-300 cm from ICV); given IFX, UST, or vedolizumab only
**Vale Rodrigues et al. (2020)^19^, Portugal, Spain, Greece, Switzerland, Germany, Italy Malta, UK, Israel, Netherlands, China**	Retrospective case series observational	Multi-center	40 adult CD, all esophageal (isolated L4–4; L1–9, L2–3, L3–24)	Endoscopic, clinical, histologic, radiologic (CT, MRE)
**Yamamoto et al. (2009)^17^, UK**	Retrospective case series observational, 12 years	Multi-center	54 adult CD, all had gastroduodenal (only 2 had exclusive UGICD; 37 had concomitant CD in small bowel alone, 8 large bowel alone, 7 both small and large bowel)	Endoscopic, radiologic, histologic
**Lui et al. (2017)^21^, USA**	Retrospective observational cohort, 4 years	Single center	39 adult CD, all with esophageal involvement; 2 with isolated L4 (L1 + L4, UGI and terminal ileum—6, L2 + L4, UGI and colon—5, L3 + L4, UGI and ileocolonic—26)	Endoscopic, radiologic, histologic
**Annunziata et al. (2012)^20^, Italy**	Prospective cohort, 12 weeks	Single center	119 adult CD, 19 with UGI (6/19 with concomitant small bowel involvement, 12/19 small bowel and colonic; 1/19 colonic)	Endoscopic (UGI—mouth to duodenum), histologic
**Decker et al. (2001)^16^, USA**	Retrospective observational, 1 month to 28 years	Single center	20 adult CD, all esophageal location (19/20 had extraesophageal CD, 13 had both UGI and lower GI involvement other than esophageal)	Endoscopic, clinical, radiographic, histologic
**Sandborn et al. (2011)^24^, USA, Australia, Austria, Belgium, Canada, Chile, Czech Republic, Estonia, Finland, Germany, Hungary, Israel, Italy, Latvia, New Zealand, Poland, Romania, Russia, Ukraine**	RCT, 6 weeks	Multi-center	438 adult CD, 13 with UGI involvement randomized to certolizumab (5), PBO (8) (120/438 terminal ileum, 126 colon, 179 ileocolonic involvement)	None given

a For non-RCT studies (ie, cohort), the duration of follow-up is the total amount of time patients were followed through their clinical course based on chart review, not necessarily treatment duration.

Abbreviations: CD, Crohn’s disease; CDAI, Crohn’s Disease Activity Index; CT, computed tomography; MRE, magnetic resonance enterography; MaRIA, magnetic resonance index of activity; PBO, placebo; TNF, tumor necrosis factor; UGICD, upper gastrointestinal Crohn’s disease; UST, ustekinumab; IFX, infliximab.

## 3. Results

### 3.1. Search results

We identified a total of 1207 citations (Figure S1). One additional record was identified through other sources. After the removal of 44 duplicates, 1163 records underwent initial screening based on titles and abstracts. Of these, 130 full-text articles were retrieved and reviewed for eligibility. A total of 117 articles were excluded, with detailed reasons (Table 2). Thirteen studies met our inclusion criteria, comprising 11 observational studies (eight retrospective cohorts and three prospective cohorts), and two post-hoc analysis of RCTs. No study exclusively compared controlled interventions for UGICD patients.

**Table 2.** Treatment interventions and outcomes.

Author, year	UGI subset, age group	Study design, duration of follow-up	Interventions studied	Outcomes measured	Key numeric results	Authors’ main interpretations related to UGICD
**D’Haens et al. [DIVERGENCE 1]. (2023)^25^**	17 adults, jejununal	RCT, 24 weeks	Filgotinib, placebo	MARiAseg remission (MaRIAseg of <7 in segments with baseline MaRIAseg of 7) at 24 weeks^25^	**Non-significant.** (MARiAseg mean): 0/3 PBO (6.6); 0/8 FIL 100 mg (6.3); 2/6 FIL 200 mg (6.8)^25^	Filgontinib did not result in statistically significant differences vs PBO to achieve MARiAseg remission in jejunum in 24 weeks.^25^
**D’Haens et al. (1994)^14^**	14 adults, esophageal	Retrospective observational, 12 years	“Usual therapy”: Steroids ± 5-ASA ± Abx, immunomodulator ± acid suppression, no uniform prospective regimen	Endoscopic healing, flares/relapse at follow-up	**Significant.** 69% (9/13) 64.3% (9/14) with partial or complete healing after short course steroids; 21% (3/14) had persistent disease or repeated flares.^14^	Esophageal CD responds to steroids in ∼70%.^14^
**Miehsler et al. (2001)^15^**	32 adults, UGI	Retrospective observational, median 13.5 months	4 arms: PPI, 5-ASA, steroid, AZA	CDAI, UGI symptom score, CRP	**Significant.** Antisecretory drugs: UGI symptom score 2.5 (1-5) to 1 (0-3) *P *= 0.039 Prednisolone: CDAI from 272 (151-451) to 100 (22-309) *P *= .03 AZA: CDAI 94 (39-426) to 57 (0-203) *P *= .04; CRP 2.3 mg/dL (0.5-8.2) to 0.7 mg/dL (0.5-2.2) *P *= .02^15^	UGI symptoms responded to antisecretory drugs. CDAI decreased with prednisolone, AZA. CRP decreased with AZA.^15^
**Olbjorn et al. (2014)^23^**	16 pediatric, UGI	Prospective cohort, 2 years	IFX after failure of standard treatment with EEN or corticosteroids and/or 5-ASA	Endoscopic healing (defined as disappearance of ulcerations, multiple erosions, bleeding, and friability. Both complete normalization (grade 0) and light hyperemia and granularity (grade 1) were considered as endoscopic healing), clinical remission (PCDAI<10) maintained at follow up at 1-2 years^23^	**Significant.** IFX treated: from 11/16 with UGICD-specific lesions at diagnosis to 2/16 at follow-up (69% to 13%); non-IFX treated: 5/16 at dx to 2/16 at follow-up (31% to 13%)^23^	IFX resulted in a significant decrease in UGICD-specific lesions at follow-up 1-2 years after dx. Response was sustained at 2 years for all responders.^23^
**De Felice et al. (2015)^6^**	24 adults, esophageal (3 L4)	Retrospective observational case series, 1 year	Alone or combination: 5-ASA, prednisone, AZA, oral budesonide, IFX, CZP, PPI	Clinical and endoscopic response at 1 year. Clinical response was defined as complete (resolution of all esophageal symptoms), partial (improvement but no resolution of esophageal symptoms), and no response (worsening or no change in esophageal symptoms). Endoscopic response was defined as complete (resolution of ulcerations, strictures, and fistulas), partial (improvement of ulcerations, strictures, and fistulas), and no response (worsening or unchanged/persistent ulcerations, strictures, and fistulas).^6^	**Significant.** Patients were treated with various combinations, in this case series. Inflammatory esophageal CD—7/9 completely responded to prednisone with 1 partial response, 2 patients responded to topical budesonide; 5 patients treated with IFX monotherapy achieved clinical response. 2 responded with IFX after initial prednisone induction, and one patient who initially failed certolizumab subsequently achieved complete response after switching to IFX. 4 patients with stricturing CD—ADA ± 6-MP with varying response; 2 fistulizing CD; 1 responded to IFX after PPI, AZA, tacrolimus and oral hydrocortisone, another had no response to immunomodulators^6^	Inflammatory esophageal CD responded to prednisone, topical budesonide, or biologics. Stricturing esophageal CD was successfully treated with combination of biologic therapy, immunomodulators, and serial dilatations with/without steroid injections.^6^ Aggressive medical therapy with biologics for fistulizing esophageal CD was not universally effective.^6^
**Lambin et al. (2020)^18^**	60 adults, UGI strictures	Retrospective observational, 5 years	Immunomodulator (AZA, MTX), anti-TNF, anti-TNF + immunomodulator, none, UST	Primary outcome: surgery free-survival at 1 and 5 years. Secondary: clinical efficacy of medical, endoscopic and surgical treatments, defined by an 8-point improvement of obstructive symptoms according to physician global assessment, (2) immediate endoscopic efficacy of endoscopic treatment defined by a stricture passable by a standard endoscope after the procedure^18^	**Significant.** Anti-TNF alone: OR 0.2, 95% CI: 0.04-0.9; *PP *= .03; 70% efficacy at 6.5 months anti-TNF + immunomodulators: OR 0.2; 95% CI: 0.05-0.9; *PP *= .03; 61% efficacy at 23.5 months UST + immunomodulator: 67% efficacy at 10.5 months UST alone: 50% efficacy, Immunomodulator alone: 50% improvement in 35mos.^18^	Anti-TNF therapy alone or in association with an immunosuppressant reduces risk of surgery in UGICD strictures.^18^
**Huang et al. (2025)^22^**	39 adults, jejunum	Retrospective observational cohort, 1 year	IFX, UST, vedolizumab	Endoscopic healing at 1 year (defined as a modified SES-CD of 0 in the segment assessed)^22^	**Significant.** After 1 year: jejunal endoscopic healing (EH): IFX 8/15 (53.3%), UST 3/15 (23.1%), vedolizumab 1/11 (9.1%) Endoscopic response (ER): IFX 8/15 (53.3%), UST 7/13 (53.8%), vedolizumab 2/11 (18.2%) Ulcer healing: IFX 8/15 (53.3%), UST 5/13 (38.5%), vedolizumab 1/11 (9.1%)^22^	IFX had strongest response compared to either UST or vedolizumab for endoscopic healing, endoscopic response, and ulcer healing in jejunum. Primary outcome at 1 year for all 3 biologics in jejunum: 30.8% endoscopic healing (EH), 43.6% achieved endoscopic response (ER), 35.9% had ulcer healing (UH)^22^
**Vale Rodrigues et al. (2020)^19^**	40 adults, esophageal	Retrospective observational	PPI, corticosteroids, 5-ASA, anti-TNF, thiopurines, EEN	Clinical and endoscopic remission	**Significant.** 37/40 (92.5%) tx with PPI; 33/40 (82.5%) treated with >1 drug; 33/40 (82.5%) with 1st line therapy had complete resolution in 7 months^19^	Clinical and endoscopic remission was achieved in more than 80% of the patients, majority with PPIs.^19^
**Yamamoto et al. (2009)^17^**	54 adults, gastroduodenal CD	Retrospective observational, 12 years	Medical therapy in 31/54: corticosteroids, 5-ASA, AZA, PPI alone or combination	Clinical response; need for surgery	**Nonsignificant.** 14/31 (45%) responded to medical tx; 19/31 required surgery, 12 did not require surgery^17^	Medical therapy not effective for UGICD, although corticosteroids most widely used.^17^
**Lui et al. (2017)^21^**	39 adults, esophageal	Retrospective observational, 4 years	Anti-TNF, anti-integrin, anti-IL12/23, combination	Clinical response/remission, endoscopic response/remission (defined as complete resolution of symptoms and baseline endoscopic findings)^21^	**Significant.** Anti-TNF resulted in greater clinical (*P *= .02) & endoscopic (*PP *< .01) response and clinical remission (*P *= .01) but not endoscopic remission (*P *= .85); anti-integrin poorer endoscopic response when tx with other biologics (*P *< .03) & not assoc. with significant clinical response; anti-IL12/23 compared with other biologics associated with poorer clinical (*P *< .01) and endoscopic response (*P *= .03); no significant difference between anti-integrin and IL12/23 clin response (*P *= .13), clinical remission (*P *= .34), endoscopic response (*P *> .99), and endoscopic remission (*P *> .99).^21^	The timing of biologic initiation in patients in this study was not significantly associated with improved clinical or endoscopic outcomes. Treatment with anti-TNF therapy, when given as first-line treatment associated with better clinical and endoscopic outcomes when compared to other second- or third-line biologic therapies.^21^
**Annunziata et al. (2012)^20^**	19 adults, UGI	Prospective cohort, 12 weeks	Group A: PPI + anti-TNF (IFX, ADA) + 5-ASA, AZA, 6MP; group B: PPI + 5-ASA, AZA, 6MP	Endoscopic healing at 12 weeks	**Significant.** 100% of symptomatic patients improved. Group A: mucosal healing in 8/11 (72.7%), endoscopic healing in 7/8; Group B: 1/8 (12.5%) mucosal healing (*P *= .001)^20^	Mucosal healing achieved in 72.7% of PPI plus anti-TNF and only in 12.5% of PPI plus other treatments group.^20^
**G. Decker et al. (2001)^16^**	20 adults, esophageal	Retrospective observational, 1 month to 28 years	5-ASA, H2RA, PPI, steroids, immunomodulator	Clinical, endoscopic improvement	**Significant.** Steroids 9/18 improvement, AZA 2/8, 6-MP 2/3, PPI 2/5^16^	First-line therapy with steroids, 5-ASA, H2RA, PPI resulted in improvement in 50% of patients^16^
**Sandborn et al. (2011)^24^**	13 adults, UGI	RCT, 6 weeks	Certolizumab pegol (CZP) vs PBO with concomitant use of	Clinical remission (defined as a CDAI score ≤150 points), response at 6 weeks^24^	**Non-significant.** 1/4 CZP, 3/8 PBO^24^	CZP does not achieve statistically significant clinical remission rates at 6 weeks compared with PBO AE: headache, nausea, abdominal pain^24^

Abbreviations: ADA, adalimumab; AZA, azathioprine; 5-ASA, 5-aminosalicylate; CD, Crohn’s disease; CDAI, Crohn’s Disease Activity Index; CRP, C-reactive protein; CZP, certolizumab pegol; dx, diagnosis; EEN, exclusive enteral nutrition; FIL, filgotinib; H2RA, H2 receptor antagonist; IFX, infliximab; MaRIA, magnetic resonance index of activity; 6-MP, 6-mercaptopurine; PBO, placebo; PPI, proton pump inhibitor; RCT, randomized controlled trial; SES-CD, Simple Endoscopic Score for CD; TNF, tumor necrosis factor; tx, treatment; UGICD, upper gastrointestinal Crohn’s disease; UST, ustekinumab.

### 3.2. Description of included studies

Baseline characteristics of the 13 included studies, published between 1994 and 2025, are summarized in Table 1. Most studies (11/13, 84.6%) were observational, predominantly retrospective in design. Two post-hoc analyses of RCTs were identified. In total, 387 patients with UGICD were included. Five studies exclusively involved esophageal CD (*n* = 137), eight studies reported gastroduodenal involvement, and two studies had exclusively jejunal involvement. The majority of patients had concomitant ileocolonic disease (280/387, 72.3%); only a small subset had isolated UGICD (33/387, 8.5%). All but one study (95.9%, 371/387 patients) consisted of adult patients. Individual study sample sizes were small, ranging from 13 to 60 patients with UGICD. The mean duration of follow-up ranged from 6 weeks to 28 years. Risk of bias assessments using the NOS indicated that most observational studies had moderate to high methodological quality (Table 3).

**Table 3.** Risk of bias assessment.

Author, year	Selection				Comparability	Outcome			Total score
	Representativeness of exposed cohort	Selection of non-exposed cohort	Ascertainment of exposure	Demonstration that outcome of interest was not present at start of study		Assessment of outcome	Was follow-up long enough for outcomes to occur	Adequacy of follow-up of cohorts	
**Huang 2025**	*	–	*	*	–/–	*	*	*	6
**D’Haens 2023**	*	*	*	*	*/*	*	*	*	9
**Lambin 2020**	–	*	*	*	–/–	*	*	*	7
**ValeRodrigues 2020**	*	–	*	*	–/–	*	*	*	6
**Lui 2017**	*	*	*	*	–/–	*	*	*	7
**De Felice 2015**	*	–	*	*	–/–	*	*	*	6
**Olbjorn 2014**	*	*	*	*	–/–	*	*	*	7
**Annunziata 2012**	*	–	*	*	–/–	*	–	–	4
**Sandborn 2011**	*	*	*	*	*/*	*	*	*	9
**Yamamoto 2009**	*	*	*	*	–/–	*	*	*	7
**Decker 2001**	*	–	*	*	–/–	*	*	*	6
**Miehsler 2001**	–	*	*	*	–/–	*	*	*	7
**D’Haens 1994**	*	–	*	*	–/–	*	*	*	6

*, yes (+1 point); –, no or not applicable (0 points).

## 4. Clinical efficacy of therapeutic interventions

Given substantial variability in outcomes and patient populations across studies, results were synthesized narratively. Therapeutic efficacy and safety outcomes for each intervention are summarized below and detailed in Table 2.

### 4.1. Corticosteroids (prednisone, budesonide)

We found five retrospective cohort studies evaluating efficacy of corticosteroids. In a retrospective review of 14 adult patients with esophageal CD by D’Haens et al., 9/14 (64.3%) patients had complete endoscopic healing in 2-4 weeks with corticosteroids, while three had persistent disease or repeated flares.^14^ In a retrospective observational study by Miehsler et al. on 32 adult UGICD patients, Crohn’s Disease Activity Index (CDAI) significantly decreased from 272 to 100 with prednisolone (*P *= .03), but was not significant for improving UGI symptoms according to the UGI symptoms score (epigastric pain, pain in the stomach, heartburn, nausea, vomiting, and/or epigastric fullness, in the last 4 weeks, giving 1 point if the respective symptom was present [range 0-6]), with a median of 1.5 [range 0–4] before therapy and 1.0 on follow-up, *P* =nonsignificant [NS]) or C-reactive prtotein (CRP) with a median of 2.8 mg/dL (range, 0.4-64.2 mg/dL) before therapy and 1.7 mg/dL (range, 0.4-11.8) on follow-up (*P* = NS).^15^

De Felice et al. reviewed 24 cases of patients with UGICD, followed over a median of 4.99 years (range, 3 days to 17.7 years). Eighteen of 24 (75%) patients had inflammatory esophageal CD, and 9/18 were given prednisone, with 7/9 having complete resolution of clinical symptoms and one having partial resolution over a median therapy time of 3 months (range 26 days to 12 years).^6^ Two patients responded to topical budesonide (capsules opened and mixed with syrup).^6^ Four of 24 patients (17%) had stricturing esophageal CD, with 2/4 given prednisone and 2/4 given budesonide gel, both in conjunction with other medications (tacrolimus, dapsone, proton pump inhibitors [PPIs], infliximab [IFX], or adalimumab). Two patients who were given prednisone had no clinical or endoscopic response on follow-up at 1 year and 1/2 given budesonide gel had no clinical response but partial endoscopic response at 1 year.^6^ Two patients (8%) had fistulizing esophageal CD and both had no clinical or endoscopic response at 1 year to either beclomethasone inhaler puff and swallow or prednisone and oral hydrocortisone.

Decker et al. retrospectively reviewed 20 cases with esophageal CD and found that first-line therapy with steroids was used in 90% (18/20) of patients, with 50% (9/18) improving at the end of follow-up.^16^ However, in 17/18 patients, other medications such as PPIs, immunomodulators (azathioprine [AZA], 6-mercaptopurine [6-MP] or cyclosporine), H2 receptor antagonists (H2RAs), or 5-aminosalicylates (5-ASAs) were concomitantly given as well. They did not specify whether this improvement was clinical or endoscopic (for each patient) and follow-up duration was not uniform and ranged from 1 to 336 months.

In contrast, a retrospective study by Yamamoto et al. assessed the need for future surgery among 54 adults with UGICD. Medical therapy was initially attempted in 31/54 patients, with 77.4% (24/31) given corticosteroids, while the rest had 5-ASA, AZA, and PPIs, either alone or in combination.^17^ However, 61.2% (19/31) of patients required surgery due to obstruction or duodeno-cutaneous fistulae.^17^

### 4.2. Studies combining immunomodulators, steroids, PPIs, H2RAs and/or 5-ASA

Seven studies reported findings on combination treatments. Miehsler et al. followed 12 adults with UGICD treated with AZA while on tapering prednisone with a median dose of 10 mg. A significant decrease of CDAI (94 [range 39-426] before therapy to 57 [range 0-203] on follow-up, *P *= .04) and CRP (2.3 mg/dL [range 0.5-8.2] before therapy to 0.7 mg/dL [range 0.5-2.2] on follow-up, *P *= .02) was noted on this regimen.^15^ Lambin et al. reviewed 60 CD patients who had UGI strictures (60% [36/60] of strictures were in the duodenum). Fifty-one of 60 patients were given medical treatment, either alone or in combination.^18^ Their primary outcome was surgery-free survival at 1 and 5 years. One of the secondary outcomes was clinical efficacy, defined as improvement of obstructive symptoms (measured by physician global assessment). Immunomodulators alone were used in 21 patients (AZA in 18/21, methotrexate [MTX] in 3/21) and resulted in improvement of obstructive symptoms in 52.3% of cases (11/21) for 35 months. Immunomodulators were used with anti-tumor necrosis factor (anti-TNF) in 22 patients with clinical efficacy in 61% (13.4/22) during 23 months and with ustekinumab (UST) in three patients with clinical efficacy in 67% (2/3) during 10.5 months of follow-up.^18^

Decker et al. reported 55% (11/20) of patients who were treated with an immunomodulator (AZA, 6-MP, or cyclosporine). Two of five patients showed clinical or endoscopic improvement on AZA, two on 6-MP, and two on AZA and cyclosporine. One patient showed initial improvement on 6-MP but relapsed and needed esophagectomy. Three patients did not improve on AZA, and one did not improve on AZA followed by 6-MP.^16^ However, these 11 patients were also given other therapies in conjunction with immunomodulators. All (100%) received steroids, 9/11 (81.8%) received 5-aminosalicylates, 2/11 (18.2%) had PPIs and 2/11 (18.2%) had H2RAs.^16^ As a result, we are unable to determine individual effects of these treatments as well.

Miehsler et al. demonstrated that on a median follow-up duration of 3.5 months (range, 2-12 months), antisecretory drugs (H2RAs and PPIs) improved UGICD symptom score. Before therapy, median symptom score was 2.5 [range, 1-5]. At follow-up, it decreased to 1 [range, 0-3] (*P *= .39). However, no significant improvement was noted in either the CDAI score or CRP. Median CDAI score before therapy was 133 and decreased to 96 on follow-up (*P* = NS). Median CRP before and after therapy was 1.1 mg/dL [range, 0.4-7.3] and 1.1 [0.4-3.0] (*P* = NS), respectively.^15^ Four patients received lansoprazole and three received omeprazole for the PPI. Famotidine was used for the H2RA.

Vale Rodrigues et al. showed that among 40 adults with esophageal CD, there was complete resolution of symptoms with PPIs as first-line therapy in esophageal CD in 82.5% (33/40) of patients.^19^ However, 82% of these patients concomitantly received other treatments such as 5-ASA (40% [16/40]), systemic corticosteroids (52.5% [21/40]), anti-TNF (57.5% [23/40]), thiopurines (47.5% [19/40]), and exclusive enteral nutrition (EEN) (15% [6/40]). Only three patients were on PPI monotherapy, and of those, only one patient did not require additional therapy to achieve mucosal healing. Mucosal healing was assessed on follow-up in 29 patients and was achieved in 89.7% (26/29).^19^ Annunziata et al., on the other hand, showed that among eight UGICD adults treated with PPIs in addition to 5-ASA, AZA, and/or 6-MP, only 12.5% (1/8) achieved mucosal healing.^20^ Additionally, De Felice et al. found that 5-ASA, prednisone, AZA, oral budesonide, and PPIs were used in combination for stricturing (four patients) and fistulizing esophageal CD (one patient), but none responded clinically or endoscopically after 1 year.^6^

In addition, Lui et al. found that the timing of initiation of treatment of biologic therapy before the diagnosis of esophageal CD (given otherwise for extraesophageal or ileocolonic location) was not associated with improved clinical (87.5% vs 100%; *P *= .17) or endoscopic response (78.6% vs 90.0%; *P *= .46) when compared to outcomes in patients who were biologic naïve upon diagnosis of esophageal CD. Lastly, they found no significant difference between clinical (90.5% vs 94.1%; *P *= .67) and endoscopic response (76.9% vs 90.9%; *P *= .36) for esophageal CD with the use of combination therapy (biologics plus immunomodulator) compared to biologic monotherapy, respectively.^21^

### 4.3. Anti-TNF

#### 4.3.1. Infliximab, adalimumab

Six observational studies involving 266 patients evaluated the efficacy of anti-TNF therapy, primarily IFX, for UGICD. While none were RCTs, three studies were prospective. Although results were heterogeneous, five studies reported significant clinical outcomes.

Two retrospective comparative studies demonstrated superiority of IFX over other biologics for UGICD. Huang et al. reported that among 39 adults with jejunal CD, IFX demonstrated higher rates of segmental endoscopic healing (53.3% [8/15]) and ulcer healing (53.3% [8/15]) at 1 year compared to UST (23.1% [3/13] and 38.5% [5/13] respectively). Vedolizumab had the lowest rates with 9.1% (1/11) for both outcomes. However, IFX did not significantly outperform UST in terms of endoscopic response at 1 year (IFX 53.3% [8/15]; UST 53.8% [7/13]), although both were superior to vedolizumab (18.2% [2/11]).^22^ Huang et al. defined endoscopic response as a greater than 50% reduction in the modified Simple Endoscopic Score for CD (SES-CD) at both the complete and segmental levels.^22^ Similarly, Lui et al. reported that among 39 adults with esophageal CD, initial treatment with anti-TNF therapy significantly improved clinical response (96.8% vs 71.4%; *P *= .02), clinical remission rates (64.5%% vs 14.2%; *P *= .01) and endoscopic response (94.7% vs 40.0%; *PP *< .01) compared to treatment with other biologics, including UST and vedolizumab. However, endoscopic remission was not significantly different between groups (63.2% vs 20.0%; *P *= .85).^21^

De Felice et al. described outcomes in 24 adults with inflammatory, stricturing, and fistulizing esophageal CD.^6^ Eighteen of 24 patients had inflammatory esophageal CD. Five of these 18 patients were treated with IFX monotherapy and all (100%) achieved clinical response. Additionally, 2/18 patients responded fully to IFX after initial prednisone induction, both without recurrence of esophageal symptoms, and one patient who initially failed certolizumab subsequently achieved complete response after switching to IFX. Four patients had stricturing esophageal CD, one of whom achieved complete response to adalimumab combined with 6-mercaptopurine, dapsone, and steroid injections. One patient had complete clinical and partial endoscopic response to adalimumab and serial dilatations, while another had no response to IFX and dilatations.^6^ On the other hand, one patient partially responded to IFX plus dapsone, PPIs, and balloon dilatation. Two patients had fistulizing CD; one achieved complete fistula healing with IFX combined with tacrolimus and PPIs after failing multiple prior treatments (including PPIs, AZA, tacrolimus, and oral hydrocortisone) while the other underwent procedural interventions without immunomodulators and had no response.^6^

Succeeding studies in our review evaluated IFX in combination with other treatments, complicating the direct attribution of clinical outcomes solely to IFX. Annunziata et al. conducted a prospective study in 19 adults with UGICD who were followed for 12 weeks. They reported mucosal healing in 72.7% (8/11) of patients treated with PPIs combined with an anti-TNF (10 with IFX, one with adalimumab) compared to only 12.5% (1/8) in patients treated with PPIs without anti-TNF. In both groups patients may have been on additional therapies including 5-ASA, AZA, or 6-MP.^20^

In a prospective study by Olbjorn et al., involving 16 pediatric patients with UGICD-specific lesions, IFX was initiated in patients who failed to achieve remission or experienced an early relapse following conventional induction therapies (corticosteroids, and/or 5-ASA or EEN).^23^ Patients treated with IFX also received concurrent AZA as maintenance therapy, and most (14/18, 78%) initially received EEN. Notably, IFX-treated patients had more aggressive disease characteristics at baseline, including higher inflammatory markers and more extensive UGICD involvement. Among patients with UGICD-specific lesions, those treated with IFX showed a reduction in lesions from diagnosis (11/16, 69%) to the 1-2-year follow-up (2/16, 13%). Patients who did not receive IFX experienced a less pronounced improvement, from 5/16 (31%) at diagnosis to 2/16 (13%) at follow-up. While the response to infliximab combined with AZA was sustained at follow-up, the small and uneven sample sizes limit the statistical strength of these conclusions.

A retrospective cohort study by Lambin et al. evaluated 60 adults with CD with non-passable symptomatic UGI strictures. The study found that initiation of anti-TNF therapy, either alone or combined with an immunomodulator (AZA, MTX), significantly reduced the risk of requiring surgical intervention within 5 years. Specifically, they showed clinical efficacy in reducing obstructive symptoms (based on the physician global assessment score) in 70% (14/20) of patients treated with anti-TNF therapy alone at a median duration of 6.5 months, and in 61% (13.4/22) of patients treated with a combination of anti-TNF and immunomodulators at a median duration of 23 months. Multivariate analysis demonstrated that both anti-TNF alone (odds ratio [OR] 0.2, 95% confidence interval [CI]: 0.04-0.9; *PP *= .03) and anti-TNF combined with immunomodulators (OR 0.2; 95% CI: 0.05-0.9; *PP *= .03) were significantly associated with a decreased risk of surgery.^18^

#### 4.3.2. Certolizumab pegol

Outcomes on patients with isolated UGICD reported in a randomized, double-blinded, placebo-controlled trial by Sandborn et al. were extracted from the original publication. This trial evaluated certolizumab pegol (CZP) in 439 adults with moderate to severely active CD who were anti-TNF naive. Thirteen patients had isolated UGICD. CZP was administered at 400 mg subcutaneously at weeks 0, 2, and 4 and followed-up at 6 weeks with the primary outcome defined as clinical remission (CDAI < 150) at 6 weeks. Results showed that 25% (1/4) of patients given CZP achieved clinical remission at 6 weeks, compared with 38% (3/8) on placebo.^24^ However, the proportion of patients who received CZP was approximately half of those who received placebo.

#### 4.3.3. IL12/23 inhibitor (ustekinumab)

Two studies reported on the efficacy of UST. A subgroup analysis of Lambin et al. (*n* = 60), involving patients with symptomatic UGI strictures, reported that UST monotherapy (*n* = 2) resulted in clinical efficacy (improvement of obstructive symptoms according to physician global assessment) in one patient (50%) during a 4-month follow-up, whereas UST combined with an immunomodulator (*n* = 3) demonstrated clinical efficacy in two patients (67%) at a median follow-up of 10.5 months.^18^ On the other hand, Lui et al.’s retrospective cohort study found that compared with other biologics (IFX and vedolizumab), UST was associated with poorer clinical (70.0% vs 100.0%, *P *< .01) and endoscopic response (57.1% vs 94.1%, *P *= .03) and there was no significant difference between anti-integrin and UST in terms of clinical response (66.7% vs 33.3%, *P *= .13), clinical remission (66.7% vs 33.3%, *P *= .34), endoscopic response (50.0% and 50.0%, *P *> .99) or endoscopic remission (50.0% and 50.0%, *P *> .99).^21^

#### 4.3.4. Anti-integrins (vedolizumab)

Two studies reported on the effects of vedolizumab on UGICD. Lui et al. showed that there was no significant difference between vedolizumab and IL12/23 in terms of clinical response (66.7% vs 33.3%, *P *= .13), clinical remission (66.7% vs 33.3%, *P *= .34), endoscopic response (50.0% vs 50.0%, *P *> .99), and endoscopic remission (50.0% vs 50.0%, *P *> .99).^21^ Vedolizumab also had poorer endoscopic response when compared to other biologics (IFX and UST combined) (57.1% vs 94.1%, *P *< .03).^21^ Similarly, a study by Huang et al. revealed that among 110 adults with proximal small bowel involvement of CD, vedolizumab was the least effective (5.5% [1/18]) in achieving endoscopic healing at 1 year compared to IFX (29.6% [19/64]) and UST (10.7% [3/28]).^22^

#### 4.3.5. Filgotinib

The DIVERGENCE 1 trial by D’Haens et al. was a randomized, double-blinded, placebo-controlled trial evaluating the efficacy of a JAK1-specific inhibitor, filgotinib (given at doses of 100 or 200 mg), in 78 CD patients with small bowel involvement.^25^ Affected segments included at least one of the following: jejunum, proximal ileum, and terminal ileum. The primary outcome was radiologic remission, defined as transmural healing measured by magnetic resonance index of activity (MaRIA) at 24 weeks.^25^ We included 17/78 patients with jejunal involvement in our review. Segment-level analysis showed that filgotinib (FIL) did not result in statistically significant differences compared to placebo (PBO) (FIL 100 mg 0% [0/8], FIL 200 mg 33.3% [2/6] vs PBO 0% [0/3]) in achieving transmural healing in the jejunum at 24 weeks. However, in this trial, only 55.1% (43/78) of patients completed the study to 24 weeks, as those who were non-responders or whose disease worsened at week 10 were discontinued and did not complete the 24-week course.

### 4.4. Risk of bias assessment

We assessed the risk of bias in all 13 studies with the NOS cohort study version. Scores for individual studies are summarized in Table 3. Each study was assessed on three domains: participant selection criteria, comparability of groups, and outcome ascertainment. For participant selection, 38.5% (4/13) had low risk of bias and 61.5% (8/13) had moderate risk of bias. For items under participant selection, 84.6% (11/13) and 53.8% (7/13) of studies had low risk of bias for representativeness of cohort and selection of non-exposed cohort, respectively. All studies showed ascertainment of exposure and demonstrated that the outcome of interest was not present at the start of the study.

For the comparability domain, only 15.4% (2/13) had low risk of bias, while 84.6% (11/13) had high risk of bias, as they were uncontrolled. For the outcome domain, 92.3% (12/13) of studies had low risk of bias, and one had high risk of bias due to a very short follow-up period for outcomes and cohorts. Overall, 61.5% (8/13) of studies included in our systematic review had high methodological quality (low risk of bias), 30.8% (4/13) had moderate quality, and one had low quality (high risk of bias).

## 5. Discussion

This comprehensive systematic review spanned 35 years of available studies from 1990 to 2025, particularly in the biologic and advanced therapy era. We summarized current available data on anti-TNF, corticosteroids, anti-IL12/23, anti-integrin, as well as combination treatment with immunomodulators, 5-ASA, PPIs, H2RAs, and EEN. However, because we did not identify well-controlled studies focused on UGICD therapy, our results highlight a void of high-quality evidence to inform therapeutic strategies in this subset of patients, who often experience substantial symptoms and poor quality of life.

Although we initially found 63 of 1207 citations which included patients with UGICD (L4) involvement, these studies did not report the effect of therapy on the UGI segment in the final analysis and were excluded, perhaps because during the time these studies were made, the estimated prevalence of UGICD was thought to be negligible; however, recent data in the last 5 years challenge this assumption.^3^^,^^4^ Furthermore, we have not found data exclusively examining the therapeutic effect of interventions such as EEN, biologics, or small molecules on UGICD.

Because most studies were retrospective and uncontrolled, physicians probably treated patients based on their overall disease burden, rather than UGI involvement alone. However, recent studies suggest that CD involving the more proximal segments of the GI tract should be considered a distinct phenotype, as UGI and proximal small bowel CD appear to be more aggressive and take longer to achieve endoscopic or radiologic healing compared to ileocolonic phenotype (with small bowel healing usually occurring only after 9-12 months of therapy compared to 6-9 months in colonic disease).^25–28^ Therefore, one should be cautious about the generalizability of the efficacy of available therapies used in ileocolonic CD in patients with UGI tract involvement. In our review, 72.3% of patients involved in the included studies had extraesophageal or ileocolonic involvement.

Despite the limited evidence in our review, IFX showed the most favorable outcomes for UGICD. These findings are derived from small observational studies. Interpretation of data may also be skewed due to use of concomitant treatments. Two recent small retrospective cohort studies published within the last 3 years specifically showed that IFX had superior efficacy compared to UST and vedolizumab in achieving clinical response, clinical remission, and endoscopic response, though not endoscopic remission at 1 year.^21^^,^^22^ These results align with the findings of Cohen et al.’s systematic review of treatments for UGICD.^5^ Notably, individual comparative efficacy data for other anti-TNF agents, such as adalimumab and certolizumab, were not explicitly provided, as all six studies reviewed grouped these biologics collectively with IFX. Additionally, certolizumab was found to be ineffective for clinical response in UGICD in one study, though this result was limited by a short follow-up period of only 6 weeks.

Similar to ileocolonic CD, a systematic review and meta-analysis indirectly comparing IFX and vedolizumab in moderate to severe CD showed that IFX was more efficacious than vedolizumab, resulting in better CDAI or clinical response in the induction phase, but not the maintenance phase.^29^ In Lui et al.’s study, vedolizumab was found to be the least potent among the three biologics, which seems to mirror what we observe for ileocolic disease.

Our review also showed that the efficacy of steroids is more heterogeneous than that of anti-TNF, with five studies all with a moderate to low risk of bias. Traditionally, corticosteroids have been used in the treatment of active CD, so it is expected that corticosteroids would be uniformly efficacious in treating UGICD as well. However, it appears that from our review, steroids helped achieve endoscopic response for inflammatory lesions of UGICD but not for stricturing or fistulizing disease. In addition, a retrospective observation by Miehsler et al. demonstrated a significant decrease in CDAI from 272 to 100 with prednisolone (*PP *= .03), without significant improvement in UGI symptoms or achieving clinical remission.^15^ Of note, CDAI has only been validated for ileocolic CD.^30^

PPIs are believed to facilitate mucosal healing by acid suppression, and ECCO guidelines recommend PPIs for mild esophageal CD, reserving corticosteroids and anti-TNF for more severe cases.^31^ However, the true individual effects of drugs such as PPIs, H2RAs, immunomodulators, 5-ASA, and EEN cannot be reliably estimated from our review, as many of the studies analyzed these therapies in combination rather than as monotherapy. Additionally, caution is warranted regarding long-term PPI use in CD patients—particularly when combined with non-steroidal anti-inflammatory drug (NSAIDs)—as PPIs have been associated with increased prevalence of NSAID-related small-bowel injury (OR ≈ 3.0), probably mediated via gut microbiota alterations and dysbiosis, and are linked with reduced microbial diversity and worse clinical outcomes in IBD cohorts.^32^^,^^33^

Across all studies, there was significant variability in how response or remission to therapy was defined. Clinical response was assessed using the CDAI, Pediatric CDAI, Lewis score, or UGI symptom score. No standardized, validated measures for endoscopic or radiologic response or remission were consistently used. For example, D’Haens’ DIVERGENCE trial used MaRIA score, which was not previously a primary outcome, possibly limiting observed treatment effects. No standard follow-up period exists for endoscopic, clinical, or radiologic response in UGICD; the DIVERGENCE trial’s 24-week follow-up may be too short, as transmural healing can take 9-12 months. Patients who did not respond or worsened were discontinued at 10 weeks, possibly underpowering the study. CZP showed no effect in Sandborn et al.’s small trial, unlike IFX, but response was only measured after 6 weeks. Both studies indicated non-significant efficacy for UGICD patients.

A major barrier to clinical trial design in UGICD is the lack of validated, disease-specific endpoints. Existing indices such as CDAI or SES-CD were developed for ileocolonic disease and may not capture the unique clinical spectrum of UGICD. We propose that future trials incorporate co-primary endpoints including endoscopic or radiologic healing (with adapted segmental SES-CD or MaRIA scoring) and patient-reported outcomes (PROs) reflecting UGI symptom domains (eg, dysphagia, odynophagia, epigastric pain, obstructive symptoms). In the absence of validated tools, modification of existing indices coupled with structured symptom assessments may serve as an interim strategy until dedicated UGICD PROs are developed and validated.

Similar to ileo-colic CD, UGICD has different phenotypic presentations—inflammatory, stricturing, and fistulizing—with varying treatment efficacy as shown by De Felice et al.^16^ Inflammatory and stricturing CD appear to be respond better to therapies such as anti-TNF, but data on fistulizing UGICD are limited. Fistulizing ileocolonic disease is more aggressive, requiring high doses of IFX.^31^^,^^34^ Strictures have been reported in 71% of UGICD cases.^35^ Lambin et al. observed a reduced surgery risk within 5 years in 60 adults with non-passable symptomatic UGI strictures, especially with anti-TNF therapy combined with an immunomodulator.^18^

The most common sites of involvement for CD are the ileal and colonic segments of the GI tract, with an estimated 26% and 35% prevalence, respectively.^36^ Until the last 5 years, involvement of the UGI tract was thought to have been rare and associated more with the pediatric population, so it is unsurprising that this phenotype has been underrepresented in the overwhelming majority of clinical trials for adult CD in the last 30 years. In addition, esophagogastroduodenoscopy is not routinely performed in adults diagnosed with CD. These factors potentially lead to missed cases. However, upstream disease may be potentially detected with imaging with baseline computed tomography or magnetic resonance enterography, according to ECCO or American Gastroenterological Association (AGA) guidelines.^31^^,^^37^

A key limitation of our review was the substantial variability in outcome definitions used across studies, complicating direct comparisons. Clinical responses were inconsistently assessed using indices such as CDAI, Pediatric CDAI, Lewis score, or non-standardized symptom scales, none validated specifically for UGICD. Likewise, no standardized definitions for endoscopic or radiologic outcomes exist. Moreover, an established definition of UGICD itself is lacking. We defined UGICD to include jejunal involvement—unlike previous studies restricted to the duodenum—based on phenotypic similarity with esophageal and duodenal CD. Our inclusion of jejunal disease reflects growing recognition that CD affecting the proximal small bowel may represent a distinct phenotype, with outcomes that differ from ileocolonic disease. Because definitions of the jejuno-ileal transition vary across studies and guidelines, we interpreted jejunal involvement primarily using endoscopic landmarks (ligament of Treitz proximally, jejuno-ileal junction distally), supported by radiologic findings when available, rather than relying on rigid distance-based measurements, which are operator- and device-dependent. For transparency, we preserved the definitions reported by individual studies (eg, Huang et al.) but interpreted these within this broader anatomic framework. This approach acknowledges the definitional controversy while providing a consistent basis for including jejunal disease in our analysis. Studies limited to esophageal involvement may reduce generalizability to broader UGICD populations. Historically, patients with UGICD have been excluded from trials due to difficulties assessing endoscopic outcomes, as current scoring tools target ileocolonic disease. The CDAI may also inadequately capture unique UGI symptoms such as dysphagia or obstruction. These issues underscore the need to develop standardized, UGI-specific outcome measures and symptom assessment tools to enable more meaningful evaluation in future clinical trials.

Although nutritional therapy may be relevant in UGICD due to frequent dietary intolerance and weight loss, no eligible studies specifically addressing dietary interventions were identified. Patients commonly modify their diet empirically, and defined diets (eg, Crohn’s Disease Exclusion Diet, Specific Carbohydrate Diet) have shown potential in general CD populations.^38^^,^^39^ Clinicians may extrapolate these approaches to UGICD, but formal evidence is currently lacking.

In conclusion, our review highlights the large unmet need for controlled studies or trials dedicated for UGICD, as well as standardized definition and treatment goals. The myriads of emerging biologic and small molecule therapies and their combinations may present an even greater opportunity to examine efficacy of these novel therapies in years to come.

## Supplementary Material

jjaf187_Supplementary_Data

## Data Availability

All data underlying this article are available within the manuscript and its supplementary material. No new data were generated beyond the published studies cited.
